# Urban physical food environments drive dietary behaviours in Ghana and Kenya: A photovoice study

**DOI:** 10.1016/j.healthplace.2021.102647

**Published:** 2021-09

**Authors:** Rebecca Pradeilles, Ana Irache, Milkah N. Wanjohi, Michelle Holdsworth, Amos Laar, Francis Zotor, Akua Tandoh, Senam Klomegah, Fiona Graham, Stella K. Muthuri, Elizabeth W. Kimani-Murage, Nathaniel Coleman, Mark A. Green, Hibbah Araba Osei-Kwasi, Marco Bohr, Emily K. Rousham, Gershim Asiki, Robert Akparibo, Kobby Mensah, Richmond Aryeetey, Nicolas Bricas, Paula Griffiths

**Affiliations:** a School of Sport, Exercise and Health Sciences, Loughborough University, United Kingdom; b Warwick Medical School, University of Warwick, Coventry, UK; c African Population and Health Research Center, Nairobi, Kenya; d IRD (French National Research Institute for Sustainable Development), NUTRIPASS Unit, Université de Montpellier-IRD, Montpellier, France; e University of Ghana, Department of Population, Family & Reproductive Health, School of Public Health, Accra, Ghana; f Department of Family and Community Health, School of Public Health, University of Health and Allied Sciences, Ho, Ghana; g Population Health Sciences Institute, Newcastle University, United Kingdom; h Department of Geography and Planning, University of Liverpool, UK; i Department of Geography, University of Sheffield, Sheffield, UK; j School of Art & Design, Nottingham Trent University, UK; k Public Health Section, School of Health and Related Research, University of Sheffield, United Kingdom; l Department of Marketing and Entrepreneurship, University of Ghana Business School, Accra, Ghana; m UMR MOISA, CIRAD- Agricultural Research & International Cooperation Organization, Montpellier, France; n Department of Obstetrics and Gynaecology, University of Ghana Medical School, College of Health Sciences, Accra, Ghana

**Keywords:** Dietary behaviours, Physical food environment, Food safety, Photovoice, Ghana, Kenya

## Abstract

We identified factors in the physical food environment that influence dietary behaviours among low-income dwellers in three African cities (Nairobi, Accra, Ho). We used Photovoice with 142 males/females (≥13 years). In the neighbourhood environment, poor hygiene, environmental sanitation, food contamination and adulteration were key concerns. Economic access was perceived as a major barrier to accessing nutritionally safe and healthy foods. Home gardening supplemented household nutritional needs, particularly in Nairobi. Policies to enhance food safety in neighbourhood environments are required. Home gardening, food pricing policies and social protection schemes could reduce financial barriers to safe and healthy diets.

## Highlights

• Food safety, physical distance and affordability were important influencers of diet.
• Home gardening helps make healthy, safe food more accessible to households.
• Economic access was perceived as a major barrier to accessing nutritionally safe and healthy foods.
• Policies to enhance food safety in neighbourhood environments are required.
• Food pricing policy/social protection could make healthy, safe foods more affordable.

## 1 Introduction

Low-and middle-income countries (LMICs), including countries in Sub-Saharan Africa (SSA), are experiencing dietary transitions as a result of food system transformations following socio-economic development (Imamura et al., 2015; Popkin et al., 2020). Changes in diet towards the consumption of energy-dense nutrient-poor (EDNP) foods, rich in saturated fat and added sugars, combined with low levels of physical activity have significantly contributed to a rise in overweight/obesity and nutrition-related non-communicable diseases (NR-NCDs), such as type 2 diabetes and cardiovascular diseases (Abrahams et al., 2011; Popkin et al., 2012; Branca et al., 2019). Consequently, unresolved forms of undernutrition (stunting, wasting and micronutrient deficiencies) now coexist with NR-NCDs in most LMICs at the individual, household, and/or country-level (Global Nutrition Report, 2020; Popkin et al., 2020).

There has been a significant rise in overweight and obesity among both men and women in SSA countries (Global Nutrition Report, 2020) and its prevalence is twice as high among urban dwelling women compared to men (Amugsi et al., 2017). The overall prevalence of overweight and obesity in Ghana increased from 29.3% (women) and 14.4% (men) in 2000 to 41.0% (women) and 22.1% (men) in 2016. In Kenya, overweight/obesity increased from 22.5% (women) and 11.6% (men) in 2000 to 32.6% (women) and 16.3% (men) in 2016 (Global Nutrition Report Country Profiles, 2019a; 2019b). Similarly, in children and adolescents (5–19 years old), the prevalence of overweight and obesity has steadily risen from 5.5% (Ghana) and 4.9% (Kenya) in 2000 to 10.7% (Ghana) and 11.2% (Kenya) in 2016 (Global Nutrition Report Country Profiles, 2019a; 2019b). Additionally, evidence from middle-income countries, such as Ghana and Kenya, suggests less affluent women are more likely to present obesity, but this is mixed for men (Dinsa et al., 2012). Non-communicable diseases have become an important public health issue in both countries accounting for 43% (Ghana), and 27% (Kenya) of total deaths (World Health Organization, 2018a, 2018b).

In view of this new nutrition reality, there is an increasing interest in enabling healthier dietary behaviours through creating healthier food environments (Hawkes et al., 2020). This includes ensuring that healthy foods/beverages are available, accessible (physically or financially) and appealing, while discouraging the consumption of ultra-processed or EDNP foods. Additionally, understanding factors that drive dietary behaviour are important for designing relevant policies and interventions to enhance food environments (Global Nutrition Report, 2020). Most existing research has focused on individual level factors, so little is known about the physical food environmental factors driving dietary behaviours in urban Africa (Gissing et al., 2017; Green et al., 2020; Osei-Kwasi et al., 2020).

The aim of this paper was to identify, through the use of participatory photography (Photovoice), factors in the physical food environment that influence dietary behaviours (defined as a combination of eating habits, preferences, choices and feeding-related mannerisms (Stok et al., 2017)) among urban dwellers in three African cities.

## 2 Material and methods

### 2.1 Study setting

This study was part of a wider project (Holdsworth et al., 2020) conducted in three rapidly growing urban African cities in Ghana (Accra (capital city, population ~2.5 million) and Ho (secondary city, population ~100 000)) and Kenya (Nairobi (capital city, population ~ 4 million)). Although Ghana's population is highly urbanised (>50%), only about a quarter of Kenya's population live in urban settings. The three cities targeted present with differing levels of urbanisation and overweight/obesity prevalence. Accra/Ho (Ghana) and Nairobi (Kenya) were selected based on differences in overweight/obesity prevalence in the regions comprising each city. At study conception (2015), the prevalence of overweight and obesity was 57.3% (females) and 29.9% (males) in Greater Accra, 31.1% (females) and 10.2% (males) in the Volta region (Ho) (Ghana Statistical Service (2015)) and 47.6% (females) and 17.3% (males) in Nairobi (Ettarh et al., 2013; Kenya DHS, 2014).

### 2.2 Study design

A qualitative study design was used to investigate the factors in the physical food environment that are perceived to influence dietary behaviours. To address the aim of this study, a community-based participatory photography method (Photovoice) was adopted. This photo-elicitation technique, whereby participants use cameras to document their daily lives and the communities where they live, allowed an in-depth exploration of the factors that are perceived to influence dietary behaviours. This method has previously been employed in resource-poor settings of high-income countries (Heidelberger and Smith, 2015; Belon et al., 2016; Díez et al., 2017; Gravina et al., 2020) and LMICs (Auma et al., 2020; Trübswasser et al., 2020) to explore drivers of dietary behaviours. The photographs can stimulate reflection and discussion among participants and policymakers, with the intention of fostering social change (Wang and Burris, 1997; Budig et al., 2018).

This methodology was initially developed with three main goals: (i) to enable people to record and reflect their community's strengths and concerns, (ii) to promote critical dialogue and knowledge about relevant issues through discussion of photographs and hence raise awareness of complex public health issues, and (iii) to reach policymakers with the ultimate aim of improving communities in which people live (Wang and Burris, 1997; Wang, 1999).

### 2.3 Sampling and data collection

#### 2.3.1 Sampling

As this study focussed on lower wealth groups, a list of all deprived neighbourhoods in the selected cities (excluding slums) was compiled. This list was further restricted by retaining neighbourhoods that were deemed to be safe to work in by the research team. One neighbourhood in each city was then randomly selected using a manual lottery method: James Town (Accra), Dome (Ho) and Makadara constituency (Nairobi). Further details on the sampling of neighbourhoods are provided in Supplementary Appendix 1.

Within the selected neighbourhoods, participants were purposefully recruited using quota sampling based on key characteristics (i.e. age, gender, body mass index (BMI), socio-economic level, education level and occupation status) (Supplementary Appendix 2). This was to ensure breadth in the range of views, perspectives, and environments that participants were exposed to. The Photovoice study was carried out on a random sub-sample (i.e. a third) of the overall study population of the wider project (target sample: n = 64 in Accra, n = 32 in Ho and n = 48 in Nairobi; total n = 144). Recruitment took place through the communities, schools and health services. Additional information on the recruitment strategy can be found in Supplementary Appendix 3.

#### 2.3.2 Data collection

The format of the Photovoice tool used to collect data was an adaptation of the conventional format proposed by Wang (1999). This was for logistical reasons to suit the research aims, the budget available, timeframe and sample required. The main adaptation that was made for this project was to conduct one-to-one interviews instead of the more collective workshop or focus group discussion approach that is normally used in Photovoice in which participants tell their photo stories. The individual approach was used because our initial community engagement activities suggested that women in these communities were busy with work and as such it would: 1) be hard to bring women together at the same time, and 2) in some communities, the safety of group gatherings was considered a problem (at the time of the research, Kenya was experiencing political instability). Whilst recognising that this reduced the community element of engagement from the start of the process, we needed to be guided by local knowledge and opinions in designing the research. The community element came back into the research through the exhibitions.

The Photovoice tool was piloted and amendments were made to the interview guide. The initial tool was considered too long and complex for participants to respond to and resulted in a lack of depth in the content of the responses. A simplified version was compiled that focussed on the most pertinent questions to the aim of the research. In the revised version, participants were asked to take at least one photograph for each of the following topic: i. “a place where you eat and/or drink”; ii. “something/situation that makes eating healthily difficult for you”; iii. “something/situation that makes eating healthily easy for you”; iv. “something/situation that influences what you eat in your area”; and v. “a person that influences your food or drink choices in your area”. A decision was made not to define the concept of “eating healthily” for participants as we were interested in how participants defined this. The interview guide was translated into the local languages by an expert translator and then back translated to ensure none of the meaning had been lost (Supplementary Appendix 4).

Data collection took place between May 2017 and June 2018 with a total of three interactions between the research team and participants. The research team was composed of nationals who were native speakers but not members of the targeted communities. Community mobilisation happened before the research commenced as meetings were organised with gatekeepers to discuss the research proposed. These community leaders facilitated data collection and community engagement.

Participants individually attended an initial meeting with the research team to introduce them to Photovoice, train them in using the digital cameras provided and discuss ethics and safety issues (Supplementary Appendix 5). Three days after the first visit, in-person follow-ups were conducted to check on participants' progress and whether any issues had arisen with the activity. On average, the research team returned to the participant one week after the first visit to retrieve the camera memory card and print photographs for the interview. Follow-up interviews were conducted in either English or in the local languages. At the start of the interview, participants were asked to select only five photographs they would like to discuss (one per topic). We asked participants to select the most important one to them within each topic and briefly explain their choice. For each selected picture, participants were asked: i. “can you tell me what this picture shows?” and ii. “can you tell me why this picture is important to understanding your food choices in your daily life?“. During interviews, participants told the ‘stories’ of the five photographs they had taken and were asked to provide a short caption to describe their pictures. At the end of the interview, participants were also asked to choose one photograph that would appear in an exhibition aimed at telling a story about the food and drink environment in their community. Interviews were digitally recorded, lasting 45–60 min. The interviews were translated (only for those in the local languages) and/or transcribed verbatim by different members of the research team and reviewed for accuracy. Participants were given the cameras as compensation for the time spent in the research process and to empower them to use the cameras to advocate for change in other areas in the future.

### 2.4 Data analysis and synthesis

The approach taken for analysis was both theory-driven (i.e. a priori themes compiled using socio-ecological models of dietary behaviours) and data-driven (i.e. grounded codes/emerging themes from the data).

Socio-ecological models of dietary behaviours summarising evidence from high-income countries (U.S) (Story et al., 2008) and LMICs (urban SSA) (Gissing et al., 2017) were first reviewed to inform the codebook. These acknowledge the interactions between people and their environment and highlight the factors driving dietary behaviours across four levels: i. individual (e.g., preferences, knowledge, socio-demographic characteristics); social (e.g., family, friends and peers); physical (refers to environments in which people eat or source food, including the home, neighbourhood, workplace and schools) and macro (e.g., food marketing, food production and distribution systems) (Story et al., 2008). Thematic analysis was then undertaken to further complement the codebook (Braun and Clarke, 2006). Themes, defined as concepts that occurred frequently across interviews, were identified from the analysis of the coded text. Additional nodes/codes that represented emerging themes not covered in the theoretical framework were added to the original codebook, constituting the grounded codes (Supplementary Appendix 6).

The agreed codebook was used by all coders (RP/AT/SK/FG/AI/MNW) for all transcripts using NVivo version 11, which helped ensure the consistency and accuracy of the analysis across researchers involved in coding (Fonteyn et al., 2008). Additionally, all coders were extensively trained and blind double coding of 25% of the transcripts was performed to ensure consistency in applying the codebook. Any discrepancies identified during the coding process were discussed to achieve consensus.

For data synthesis, framework matrices were developed for each level of the socio-ecological framework (i.e. individual, social, physical and macro-environment) as a way of managing and summarising data (Gale et al., 2013). The final output used for interpreting and synthesising data was a summary matrix with nodes on the rows and excerpts by study sites (Accra, Ho and Nairobi) as columns to compare themes between the three cities. This paper only focuses on the findings in the physical-level environment.

### 2.5 Dissemination of findings to stakeholders through photography exhibitions

After all interviews had been completed, a photography exhibition focusing on the drivers of dietary behaviours was displayed in a public venue located at the centre of the community in each city. The purpose was to raise awareness in communities and in the media about this topic. Every participant who had given consent (>90%) had their chosen photograph and associated caption displayed at the exhibition. We produced the photographs on cloth, through local suppliers, to enable the exhibition to be recycled and used in other spaces in the future.

Local/national media and a range of stakeholders (e.g., Photovoice participants, local health and nutrition representatives, community leaders, local community group members, NGOs and representatives from government institutions) were invited to attend the opening event. At each exhibition opening, local refreshments sourced through community vendors were served and local music or dance groups were invited to perform. A dialogue was facilitated by one member of the research team to discuss the ‘story’ from the photographs with stakeholders and to raise awareness of the issues raised by the participants through their photographs.

## 3 Results

The Photovoice activity was carried out with 142 participants (out of the 144 initially targeted) across the three cities (n = 62 in Accra, n = 32 in Ho and n = 48 in Nairobi). Overall, 68.3% of participants were female and nearly half were 19–49 years old; 35.2% of participants were in work, 13.4% in education and 51.4% not in work or education (Table 1). The proportion of participants with a BMI≥25 kg/m^2^ (i.e. overweight/obesity category) was higher in Kenya (60.4%) than in Ghana (Accra: 48.4% and Ho: 46.9%).

**Table 1:** Socio-demographic characteristics of the sample.

	Total (n = 142)		Accra (n = 62)		Ho (n = 32)		Nairobi (n = 48)	
	n	%	n	%	n	%	n	%
**Gender**								
*Females*	97	68.3	40	64.5	32	100.0	25	52.1
*Males*	45	31.7	22	35.5	0	0.0	23	47.9
**Age**								
*13-18y*	47	33.1	20	32.3	12	37.5	15	31.3
*19-49y*	64	45.1	27	43.5	20	62.5	17	35.4
*≥ 50y*	31	21.8	15	24.2	0	0.0	16	33.3
**Socio-economic status**								
*Lowest*	77	54.2	32	51.6	16	50.0	29	60.4
*Low to middle*	65	45.8	30	48.4	16	50.0	19	39.6
**Occupation**								
*In work*	50	35.2	22	35.5	12	37.5	16	33.3
*In education*	19	13.4	8	12.9	4	12.5	7	14.6
*Not in work or education*	73	51.4	32	51.6	16	50.0	25	52.1
**Body mass index**								
*<*25 kg/m^2^	68	47.9	32	51.6	17	53.1	19	39.6
*≥*25 kg/m^2^	74	52.1	30	48.4	15	46.9	29	60.4

**Fig. 1:**
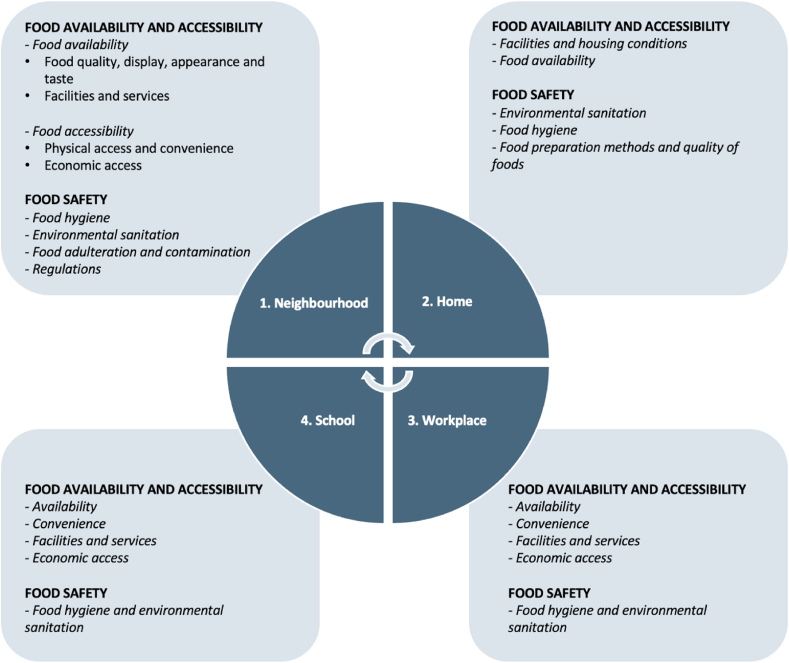
Map of the influences of dietary behaviours within the physical food environment in Ghana (Accra & Ho) and Kenya (Nairobi).

Features of the physical food environment at four different levels (neighbourhood, home, workplace and school) influenced dietary behaviours (Fig. 1); with the emerging themes organised in order of prominence (1 as most prominent) and hence an indicator of the relative contribution of the different themes towards dietary behaviours.

In the three cities, the neighbourhood food environment (food availability; economic and physical access to food and food safety) was the most prominent theme as evidenced by the extent of discussion and photographs taken. The home food environment (food availability, food safety and facilities) was also important, and emerged as integrally linked to the neighbourhood environment. There was less discussion by participants on the factors within workplaces and schools that influence dietary behaviours and hence these are not presented in detail in the manuscript. Additional information on the drivers within the workplace and school settings can be found in Supplementary Appendices 7 and 8.

### 3.1 Food availability and accessibility within the neighbourhood and home environment (theme 1)

#### 3.1.1 Sub-theme 1: food availability

In both countries, participants mentioned that food available at home influenced their dietary behaviours, e.g. whether to cook at home or buy food in their neighbourhoods (Table 2). Ultimately, this decision was made according to their food and financial situation or their family preferences.

**Table 2:** The home food environment.

Sub-theme 1: Facilities and housing conditions		

Accra	*‘That is the only place we all eat. It is our house so that is where we eat, cook and do all other things. At night, we sit by the street for some fresh air and go back inside. Unfortunately, our rooms are small in size so you cannot sit inside and eat.’ [Female, 57 years, lowest SES, A6]*	
Ho	*‘Sometimes there will be light out and then the room will be hot, so we have to sit outside. And when the sun is high, the room becomes very hot.’ [Female, 13 years, lowest SES, H9]*	
**Nairobi**	*‘ … it makes it easy for me to eat what I want to eat like the radio for instance if you hear an advertisement on the radio or a commercial break you hear more about certain foods you don't know you have never heard so it will build up your curiosity so as to know what that is, there is also the TV so still same on the advertising there is phones for the people who like social media there is no time you can open a page and miss the advertisement for food somewhere like you can get big night has an offer shawarma 350 … ’ [Female, 21 years, lowest SES, N15]*	
**Nairobi**	*‘We eat comfortably when at home. Let's say there is no table then you have to hold your cup, again you have a flask there so you don't struggle so much you just take it and when you get tired you return it to the table.’ [Female, 18 years, lowest to middle SES, N7]*	
***Sub-theme 2: Environmental sanitation***		
**Accra**	*‘The toilet been situated in front of the house isn't the best. At first it wasn't there. The scent or odour that comes from it is very bad and there are times that it makes us ill … in terms of food, there are times if we prefer to eat outside and the odour coming from the toilet facility is very bad we have no choice than to go and eat inside the room because of the scent.’ [Female, 57 years, low to middle SES, A7]*	
**Ho**	*‘It shows a gutter. This place is in the house and it is dirty. Whenever I see it I don't feel like eating … when I have cooked and say I go and fetch water, and I see it, I am unable to eat. If that place is not very neat, eating becomes very difficult for me.’ [Female, 30 years, low to middle SES, Ho, H10]*	
***Sub-theme 3: Food hygiene***		
**Accra**	*‘Well it is important because I get the kind of recipes to use … what will be good for my health and the hygiene. But someone cooking outside I do not know the kind of hygiene she has … Maybe she has not washed her hands but she has been cutting onions with her dirty hands. I will be affected at the end of the day but when I am cooking at home, I will wash my hands, I will wash the vegetables, I will get the hygiene so that when I am enjoying the food I will enjoy it to the fullest.’ [Female, 19 years, low to middle SES, A31]*	
**Ho**	*‘The reason I like to eat at her place is that, when she cooks, she cleans after herself well, and you won't know anything at that place to show that she has cooked. She will sweep the whole place, she cleans the place very well. That is why I like eating at her place.’ [Female, 13 years, low to middle SES, H28]*	
**Nairobi**	*‘I prefer to eat at home because at home you can believe it is something that you have made for yourself. I believe what I have prepared myself because I know the hygiene that I have used than what has been used out there if it is food the cleanliness that I have maintained.’ [Female, 24 years, low to middle SES, N8]*	
***Sub-theme 4: Food availability***		
**Accra**	*‘what I can add is when I have enough food stuffs at home or have enough money it makes eating easy, but then when I do not have anything it is always difficult getting food to eat.’ [Female, 30 years, lowest SES, A57]*	
**Ho**	*‘Whatever is there, they (her kids) will eat. Sometimes I tell them to eat what I have and then later, say tomorrow, we get what they want … For instance, when they want to eat rice, and I don't have rice at home, I get some money and then buy the rice and come and cook it for them. But if there is food at home, I will not worry to go and buy food.’ [Female, 25 years old, low to middle SES, H5]*	
**Ho**	*‘This picture shows that this man is a farmer. When he goes to the farm, he brings food home. If he doesn't go to the farm, there will be no food from there. That is why I took this one. He brings corn, yam, he brings palm fruits, as well as cassava and firewood.’ [Female, 43 years, low to middle SES, H15]*	
**Nairobi**	*‘I chose this picture because it is my own garden where I have planted greens. So if I want greens when the rain is good I do not have a problem. I have kales, I have avocado, Yes then it is also garden where I plant maize which I can roast and kunde, I also planted sugarcane and we eat when it has grown when you look we have sugarcane, avocado, kales, kunde and maize at the same place’ [Male, 55 years, lowest SES, Nairobi, N35]*	
***Sub-theme 5: Food preparation methods and quality of foods***		
**Accra**	*‘The food sold outside is not always hygienic and it's not enough quantity for the whole family to eat and I have to spend more money to buy those foods. So if I am able to cook at home, I will be able to feed the family during the day and also get some food to also eat in the evening before we sleep and I can save some money. Also food prepared at home is healthier because you buy the best of items to use in cooking and you take your time to prepare to your taste and satisfaction. For the foods sold outside, you don't know the ingredients used in the preparation and it's not always healthy to be eating outside foods. When you cook at home, you have the health of your family in mind and so you prepare it to get the needed strength and to be healthy. This is why I always want to cook at home.’ [Female, 55 years, lowest SES, A3]*	

In the neighbourhood food environment, availability refers to the type of foods available, the characteristics of food (quality and freshness of products, food taste and aroma and appearance), and type of food stores as well as the facilities and services these offer (Table 3).

**Table 3:** Food availability and accessibility within the neighbourhood food environment.

Sub-theme 1: Food availability		

Food quality, display, appearance and taste		
**Accra**	*‘Also she keeps the place neat and she is selling the food in a glass to prevent flies contaminating the food. The food items are well placed in neat container and package leaves are well cleaned. So these are the reasons why I like to buy my food there.’ [Male, 60 years, lowest SES, Accra, A24]*	
**Ho**	*‘The reason I took a picture of this place, this shows that all the things that the woman sells has to show, it is good, that is why I took it. The whole place shows the bowls, the spoons, and the pepper’ [Female, 43 years, lowest SES, H3]*	
**Nairobi**	*‘when something is displayed nicely it attracts you then also when you look at the person you see that he is presentable you get the appetite for eating’ [Male, 52 years, lowest SES, N39]*	
***Facilities and services***		
**Accra**	*‘Some sellers use the same spoons for everyone whilst others use disposable spoons so I prefer those who use the disposable spoons. Furthermore, how they wash their dishes is also of importance to me. Some of them do not change the water they use to wash dishes even when the water becomes dirty. I do not go to such places. I have not had any issues with her food since I started eating there and she keeps the place very neat. She has soap and water to wash your hands after eating and napkins to clean your hands as well as tissues’ [Male, 17 years, lowest SES, A14]*	
***Sub-theme 2: Food accessibility***		
***Physical access and convenience***		
**Accra**	*‘She is close to my house. I spend only 2 s to get there and buy the food. A lot of people buy the food and her food is always hot and nice … Yes they start from 4pm to 9pm. It is sold directly opposite my house … it is a really short distance from me.’ [Female, 14 years, lowest SES, A41]*	
**Ho**	*‘To go and get food to come and eat becomes an issue, because the road is too long to go and get food and come and eat … I find it difficult to eat since the road is long … if I want to eat rice, I have to get to the market to buy it. The market too is far from where I am … going to the market makes it better, because I can get it cheaper there.’ [Female, 30 years, low to middle SES, H10]*	
**Nairobi**	*‘This is the only that sells these things near here because those other ones are far. So since, let's say this one is alone is what influences us because even if you want to eat another thing, if this one does not have, you will have to change by force, in that you are forced to change to eat the thing that is in that shop because what you want is not available.’ [Female, 18 years, lowest SES, N7]*	
***Economic access***		
**Accra**	*‘ … the cost is high and I would rather use the 2 cedis to buy banku and fish and drink a lot of water and that could take me for the whole day as I explain to you previously. You see, I need the fruit but because of the cost, I cannot afford using my 2 cedis to buy apple. Fruit is good for us but the prices do change depending on whether they are in season or not and because of that we cannot buy fruits at higher prices’ [Female, 32 years, low to middle SES, A34]*	
**Accra**	*‘Things are expensive now on the market and you cannot take 10 cedis to the market to buy things needed for food. You need about 50 to 100 cedis and since I don't have that money, it makes eating difficult for me. So not having money makes eating healthy food difficult for me.’ [Female, 39 years, low to middle SES, Accra, A36]*	
**Ho**	*‘There are times, when there is no money, we can just grind pepper and eat it raw like that. Or because there is no money, from morning or we will wait till afternoon before eating. We won't eat in the morning because the food is not much so if we eat in the morning, there won't be enough for the afternoon, and there is no money to go and buy some food.’ [Female, 24 years, lowest SES, Ho, H1]* *‘Okay, with money, when you have it then you can buy something to cook at home, and when you cook it, you can get some health from it. But if you don't have money, and you go to the roadside to buy something, let's say you have 1 cedi, that cedi cannot buy anything for you to cook at home, but you can use the 1 cedi to buy something on the roadside, and people do not take care of how they cook their food, when you eat, you can fall sick. So when you have money and you buy the foodstuff and cook at home, you will have nothing to worry about with regards to your health. So money is needed, everywhere.’ [Female, 24 years, lowest SES, Ho, H1]*	
**Nairobi**	*‘Meat it is something for luxury even if it is good to the body but it is so expensive you cannot eat it every day, once a week.’ [Male, 72 years, low to middle SES, N26]*	

##### 3.1.1.1 Food quality, display, appearance and taste

Participants discussed food quality and freshness (defined as foods free from adulteration, contamination or physical damage (e.g., fruit that are rotten) and foods cooked at home) and its importance when choosing where to purchase food/meals from. Eating fresh foods was preferred and seen as more enjoyable; even though these were sometimes perceived as more expensive, and thus, less affordable. A few participants in the three cities spoke about how food display and appearance of food/food outlets influenced their dietary behaviours, both in terms of purchasing and consumption behaviours. Taste was mostly discussed by women in Accra and to a lesser extent in Nairobi. Women in Accra preferred to buy fresh food products that are ‘nice’, ‘tasty’, ‘good’, ‘delicious’ but also ‘affordable’. On the other hand, some women in Accra attributed ‘the good taste’ to the additives that food sellers add during preparation of processed meals, but they avoided these outlets as they were perceived as unhealthy.

##### 3.1.1.2 Facilities and services

In the neighbourhood food environment, facilities that influenced participants' choice of food outlets were discussed in Accra and Nairobi, but not in Ho, and referred to: i) food vendors that gave food for free or on credit, ii) services such as packaging or food vendors that prepare the foods for their customers (e.g. peeling potatoes, cutting vegetables), iii) availability of a television or a fan, iv) sitting places in food outlets, v) soap and water to wash hands at the food store before eating, vi) napkins/tissues, vii) clean disposable spoons, and viii) parking space or children's play areas.

In the home food environment, facilities refer to housing conditions (e.g. availability of space and facilities within the home) and other assets, which influence participants' dietary behaviours and the place where they eat within the house. In Accra and Ho, some participants mentioned not having adequate space to cook or eat at home. A variety of facilities, such as eating utensils, availability of chairs/tables, availability of a cool room or a fan and entertainment facilities (e.g. TV and radio) were reported by participants in all cities to make ‘eating more enjoyable’. Participants in Accra and Ho mentioned that eating on the floor or in a warm room, made eating ‘uncomfortable’.

Urban kitchen gardening (e.g. growing vegetables) and rearing small animals/poultry, such as chickens, were unique features of food availability in Nairobi, increasing the food diversity of households. Participants used kitchen gardens to supplement their nutritional needs and reduce total household food expenditure, hence ensuring household food security.

Although kitchen gardening did not emerge in Accra or Ho, a participant in Ho mentioned that having a family member working as a farmer contributed to household food security, by bringing firewood and different food products home to cook, such as cereals, tubers and fruit.

#### 3.1.2 Sub-theme 2: food accessibility

##### 3.1.2.1 Physical access and convenience

Places from where participants primarily purchased their food items included: local food outlets (Accra, Ho and Nairobi), markets (Accra, Ho and Nairobi), chop-bars (defined as an informal eatery that typically serves freshly prepared traditional Ghanaian dishes such as banku, tz, fufu, ampesi with soups and sauces. Structure may be partially or completely enclosed but the kitchen/food preparation area is usually in sight) (Accra), supermarkets (Nairobi, only on rare occasions), restaurants (Accra, only on special occasions) and other homes in the neighbourhood (Accra).

In all cities, participants chose food outlets that were convenient in terms of opening times and proximity to home, as well as those selling a greater variety of foods or healthier options (Table 3). Participants in Accra gave accounts of a greater availability of food outlets at a shorter distance, whereas in Ho, physical access to food outlets was highlighted as a barrier to eating healthily. Women in Ho struggled to buy food from their preferred outlet because stores with affordable prices are further from their homes. Access to markets was also difficult.

For some male participants in Accra, physical access did not seem to hinder eating healthily; they either walked long distances to their preferred outlet or they used a bike or a car to get there. The distance to food outlets was not raised as a concern by participants in Nairobi. Indeed, most participants described the food outlets where they bought foods to be near their homes and hence easily accessible (e.g. food kiosks, small shops and green groceries).

##### 3.1.2.2 Economic access

In all three cities, economic access to food was one of the most prominent themes that emerged (Table 3). When participants were asked what made eating healthy difficult in their area, a large number photographed bank notes. Economic access often explained why participants bought specific foods. There was agreement between participants that finances hindered access to healthy foods, and therefore, access to a healthy diet. In Accra and Nairobi, participants identified some food products, such as meat and fish, and certain fruits (only in Accra), to be unaffordable, and only purchased if they had enough money. However, traditional foods (i.e. those associated with cultural heritage) appeared to be economically accessible in both countries. In Accra, working was regarded as a facilitator to eating healthily, whereas unemployment acted as a barrier (some participants in Accra, aged 50y or more, struggled to afford food items, as they were unemployed). In Accra and Ho, ready meals or fast food (e.g. instant noodles) appeared to be often consumed by participants (although not preferred), as they were perceived as inexpensive. In Ho, women that could not afford to buy foods skipped some meals. In Accra, participants purchased meals from restaurants the least, as they were seen as overpriced.

Although economic access appeared to be a problem, with a great majority of food outlets selling ‘overpriced’ food items, all participants interviewed seemed to have a preferred food outlet in their neighbourhoods that was more affordable.

There were mixed messages regarding the cost of eating at home vs. eating out. In Accra and Ho, women preferred to cook at home, because they found this was cheaper, however, other women stated that they did not have enough money to buy all food items and ingredients required for a meal and were therefore not able to cook at home, so they would buy ready cooked foods/meals instead.

### 3.2 Food safety within the neighbourhood and home environment (theme 2)

Food safety (i.e. food hygiene, environmental sanitation, food adulteration/contamination) in the neighbourhood and the home food environment was widely discussed in all three cities, although it was a stronger theme in Accra and Ho (Table 2, Table 4). Participants described how food safety drives their food purchasing and consumption behaviours. Food adulteration was defined as the act of intentionally altering the quality of food by the admixture or substitution of inferior substances or by the removal of some valuable ingredient.

**Table 4:** Food safety within the neighbourhood food environment.

Sub-theme 1: Food hygiene		

**Accra**	*‘She covers her food with clean materials to prevent flies. Also, when she is not selling, she tries to always clean the surrounding and the utensils. Because of that I always like to buy food at her shop so I don't get stomach problems.’ [Female, 32 years, low to middle SES, Accra, A34]*	
**Ho**	*‘The place they cook the food before bringing it here, is a very neat place and the food they cook is very good. Everything that they cook and whatever they use to cook is cleaned well … those at the road side … they don't care the things they use to prepare the food. The food and how they wash the things they use to cook … They don't wash them well and it is not healthy to eat from there … ’ [Female, 38 years, low to middle SES, H27]*	
**Nairobi**	*‘she makes sure that she has washed them for you in clean water as you watch, then she cuts for you and you leave so for me it's about cleanliness if you see me going somewhere it's because of cleanliness. Even if a place the person is dirty you would not like to go maybe you took something bad started to diarrhoea because of dirt so how will you approach that person since he is dirty himself (laughter)’ [Male, 52 years, lowest SES, N39]*	
***Sub-theme 2: Environmental sanitation***		
**Accra**	*‘ … the place is not neat [clean] and for me even if the people here cook and offer me some of the food to eat, I will not eat it …. if you cook in a place like this and sell, I will not buy food from you to eat … there are dirty rags on the ground and the place is littered with plastic rubbers … ’ [Female, 24 years, lowest SES, Accra, A38]*	
**Ho**	*‘They keep that place very well. They sell by a gutter but, when they come, they clean the gutter very well before they sell. They have glass covering all their food. And the place they give you to sit if you are eating the food there, is very neat, there is soap, to use in washing your hands. When you eat, you enjoy it, even if the food is not so nice at times, you will enjoy it because of how the place is kept. How the place is neat, makes me want to eat over there.’ [Female, 19 years, lowest SES, H4]*	
**Nairobi**	*‘ … the dust goes everywhere on the foods they cook out there. Even when you go now you will get many women there some cooking chips, others things for children bhajia but it is an open place … I don't have time to cook mandazi, chapatti it is a big process and I also go to work those ones with dirt are the same ones that I buy. I ask for five mandazis, 2 chapattis if she is not around I take 4 mandazis. I came here in the 70s, and I have eaten so it is God who protects us … ’ [Male72 years, low to middle SES, N26]*	
***Sub-theme 3: Food adulteration and contamination***		
**Accra**	*‘These instant noodles that we buy almost every evening they add all sort of artificial spices to it, the sausage and all those things are not good for our body but we cannot afford the fish. We have no choice than to eat the instant noodles and sausage.’ [Female, 38 years, lowest SES, A40]*	
**Ho**	*‘There are times when they are mixing the dough, they keep talking and saliva will get into it. And also when you eat the kenkey you can see pieces of chewing sticks in it, because they will be chewing the stick and be working, and when it gets in the dough, they just wrap the kenkey.’ [Female, 13years, low to middle SES, H28]*	
**Nairobi**	*‘Go to buy from the normal cereals (shops) from which you buy in a paper, they normally put chemicals so that it doesn't spoil quickly and stay for long in the cereal (shop). So this one is good because in supermarket people come to shop like every day and so they don't need to put such things.’ [Male, 14 years, low to middle SES, N31]*	
**Nairobi**	*‘You know some people we say they put in ‘blue band’ (a brand of margarine that some vendors add to milk, together with water or other harmful chemicals such as formalin, to preserve the milk, increase its volume and make more profit), but this one is from the cow direct … ’ [Female, 55 years, lowest SES, Nairobi, N18]*	
***Sub-theme 4: Regulations***		
**Accra**	*‘I wish the Accra Metropolitan Assembly (AMA) authorities will come and inspect where people prepare food before giving them the license to cook. In Jamestown, anyone wakes up and start to sell kenkey. Interestingly, the leaves they use to bag the kenkey is not well stored and washed. They just lie on the floor and collects dust and that could cause people to fall sick. People don't wash their hands when preparing kenkey.’ [Female, 19 years, lowest SES, A35]*	
**Nairobi**	*‘So it is good if the government knows especially in urban areas, in the rural areas there are no such problems, people cook but in the urban areas people need to be told to avoid some foods like the ones in cans so people should eat foods that are fresh because the preservatives are the ones that are bringing problems to the body since our body was not made for preservatives, so when the preservatives get to the body there is no protection for those preservatives those are poisons we add to the body that bring cancers those funny disease's’ [Male, 52 years, low to middle SES, N39]*	

#### 3.2.1 Sub-theme 1: food hygiene

Participants reported concerns about food hygiene in their neighbourhood, particularly issues regarding the cleanliness of food vendors (e.g. poor hand washing practices and not wearing a hair net when handling food) and poor food preparation methods (e.g. not cleaning the food products before cooking it or using unclean water to prepare meals). There was a strong belief from the majority of participants that foods cooked outside the home was not prepared in a hygienic way, unsafe, and thus promote transmission of diseases. Although most discourse was about negative hygiene practices adopted in neighbourhoods, participants in Accra highlighted that some food vendors covered their food once it is cooked to limit contamination.

Overall, there was a clear preference for cooking at home and eating homemade foods in all cities. Homemade meals were regarded as ‘healthier food options’ as participants were able to prepare meals ‘the way they like it’, while ensuring that hygiene practices are followed, and food products are not contaminated and of good quality (e.g. items are fresh and washed).

#### 3.2.2 Sub-theme 2: environmental sanitation

Environmental sanitation in the neighbourhood food environment, which refers to the cleanliness of food outlets and area surrounding it, was a major influence on participants’ decisions of where to purchase and eat food. Participants in the three cities were aware of the negative consequences of sourcing food from unsanitary places (e.g. contamination of food and risk of food-borne related diseases) and identified a number of outlets in their neighbourhoods not meeting sanitary standards.

Similarly, the cleanliness of spaces within the home and its surroundings was a prominent theme in Accra and Ho, particularly among pregnant women (not mentioned in Nairobi), and influenced participants’ decision on where to eat meals (e.g. inside or outside the home). Places to eat, and food that is prepared next to the gutters, rubbish, open sewers or even toilets hindered eating, due to unpleasant odours and food contamination.

The collective narratives illustrate that participants avoided eating from unhygienic and unsanitary food outlets. They did this to avoid getting sick and needing to seek medical care, because of the high costs of health care. This is a real concern to participants, who state that this would lead to financial hardship for their families. However, some participants from the lowest socio-economic backgrounds in Accra and Nairobi reported having no choice but to buy from these ‘dirty’ places due to financial constraints. In both countries, it was believed that regulations should be put in place to address this issue and benefit the community's health.

#### 3.2.3 Sub-theme 3: food adulteration and contamination

Issues of food adulteration and contamination and their impact on dietary behaviours were discussed in all three cities. Adulteration and contamination of food appeared to be a common problem in Accra and more so than in Ho. Different forms of adulteration and contamination of food included: (i) the use of ‘seasoning cubes’ (Accra) and other condiments/spices to change and enhance the flavour and appearance of meals (Accra and Nairobi); (ii) utilisation of polythene bags to sell food – participants were concerned that the materials used to manufacture polythene bags could contaminate the food and represent a potential health hazard to the consumer (Accra); (iii) food uncovered which means that flies could land easily on the food (Accra); (iv) selling food items that have expired or in bad condition/stale foods (all cities); (v) not following hygienic practices such as hand-washing when preparing food (Accra and Ho); and (vi) adding unapproved chemicals for food preservation, especially to milk (Nairobi). Participants from all cities avoided purchasing from food outlets they believed used additives or sold contaminated food. In Accra, there was a general belief that food cooked/prepared at home was better than food bought outside because of the risk of food contamination/adulteration. In Nairobi, some participants reported that food adulteration was a common problem in urban areas and in small scale vendors, and it was also thought to be among the causes of cancer in urban areas.

#### 3.2.4 Sub-theme 4: regulations

Regulations emerged as a theme in Accra and Nairobi but was not raised in Ho. Women in Accra highlighted the lack of enforcement of regulations on hygiene practices and suggested that more control was needed to ensure food safety in their communities; conversely, one female participant believed that the Accra Metropolitan Assembly was ‘doing a great job’. Overall, in Accra and Nairobi, participants highlighted the need for better enforcement of regulations and control for hygiene practices of food vendors and sanitation in general. To deal with the challenge of poor food hygiene and sanitation, participants highlighted that the city council has a duty to ensure food outlets are clean when they license them and close down those that do not meet the hygiene standards. In Nairobi, participants further recommended that the government regulate the consumption of ‘junk foods’ and foods with additives, as they were perceived as key causes of poor health in urban areas.

## 4 Discussion

### 4.1 Key findings

This study provides insights into the role of the physical food environment in influencing dietary behaviours and fills an important gap in the current body of evidence in urban Africa (Gissing et al., 2017; Holdsworth and Landais, 2019; Osei-Kwasi et al., 2020).

Some of the findings of this study are similar to those already reported in the literature, in terms of factors in the food environment that influence dietary behaviours in high income contexts (e.g. food availability, physical and economic access) (Story et al., 2008) and in Africa (e.g. housing conditions, food availability, convenience) (Gissing et al., 2017; Osei-Kwasi et al., 2020). However, strong evidence was identified for the integral role of food safety concerns, particularly related to food hygiene, food contamination/adulteration and environmental sanitation, in the physical environment, which have fed into the development of an African Food Environments framework (Osei-Kwasi et al., 2021). We found differences in the factors identified across the three cities but also broad similarities.

#### 4.1.1 Availability, physical and economic access

Our findings on food availability and accessibility are consistent with those from previous studies from LMICs. Availability of food at home and economic access were among the key influencers of dietary behaviours, which was corroborated by studies conducted in other urban poor communities of African countries (de-Graft-Aikins, 2010; Layade and Adeoye, 2014; Chakona and Shackleton, 2017; Boatemaa et al., 2018). When food was unavailable, or participants could not afford to buy foods, they would eat whatever they had at home. A coping mechanism observed in our study and that contributed to the overall food availability in the household food environment was home gardening. Although this only emerged in Kenya, home gardening has also been reported elsewhere in Ghana (Bennett-Lartey et al., 2002). For urban poor families, home gardens provide cheap access to nutritive foods and a more diverse diet, contributing to household food security (Talukder et al., 2000; Ali et al., 2008; Landon-Lane, 2011; Fraval et al., 2019). As stated in a qualitative study conducted in different towns across South Africa, food grown at home does not represent the primary food source, rather it supplements food available at home or purchased in the neighbourhood (Chakona et al., 2017). Nevertheless, for some urban households, home gardening might not be an option due to lack of space and unproductive lands (Spires et al., 2020).

A recent GIS mapping study in our sampled neighbourhoods showed that informal traditional food outlets represented 70.5%, with supermarkets representing only 0.9% of outlets (Green et al., 2020). In our Photovoice study, participants reported sourcing their foods from different places (from convenience stores to supermarkets) with variation across the three cities. Most participants agreed that local informal food vendors were commonly used for food purchasing, as these were closer to home and offered a wide range of affordable food products. This is in common with findings from other studies in Africa (Spires et al., 2020). Supermarkets in LMICs, appear to be unequally distributed, mostly concentrated in wealthier areas and located at a hard-to-reach distance from low-income communities (Battersby and Peyton, 2014; Wertheim-Heck et al., 2019), which likely explains the low use of these outlets by our participants.

In terms of food available in the neighbourhood food environment, a study conducted in three poor communities in Ghana (including James Town) reported that unhealthy food options outnumbered healthy options, creating a lack of fruits and vegetables (Boatemaa et al., 2018). This contradicts our findings, as participants expressed that they were able to find healthy food items in their neighbourhoods, although these were sometimes unaffordable. Our other study on GIS mapping in our sampled neighbourhoods also highlighted a relatively high availability of healthy foods (e.g. staples, eggs, milk, vegetables) (Green et al., 2020).

In Accra and Nairobi, economic access was a predominant theme that hindered access to a healthy diet. This is consistent with similar qualitative studies, which echoed the idea that ‘healthy food is expensive’, and thus represents a barrier to adopting healthy eating, particularly for individuals who are unemployed or have insufficient money for food consumption (Chakona and Shackleton, 2017; Boatemaa et al., 2018; Trübswasser et al., 2020). Several studies from high-income countries have shown that lower socio-economic position (SEP) restricts food choices and promotes unhealthy food consumption (Powell et al., 2009; Roberts et al., 2013; Vogel et al., 2016). Other studies from African countries have also highlighted that food prices and SEP influence dietary behaviours (i.e. individuals from low SEP tend to eat less fruits and vegetables) (Charlton et al., 2004; Steyn et al., 2011; Legwegoh, 2012; Mbochi et al., 2012; Voorend et al., 2013; Landais et al., 2014, Layade and Adeoye, 2014; Sedibe et al., 2014; Pradeilles, 2015; Legwegoh and Hovorka (2016); Mayé n et al., 2016). In our study, foods seen as unaffordable included certain fruits, meat and fish, which might explain the relatively low consumption of fruit and other healthy foods among urban populations in Ghana and Kenya (Rousham et al., 2020).

#### 4.1.2 Food safety

Food safety in neighbourhoods was perceived as a barrier to adopting healthy dietary behaviours. Street food vendors play a pivotal role in providing low-income urbanites with affordable, accessible and healthy foods, but the lack of regulation in the informal food sector means there are no food safety processes in place to protect the consumers from food-borne diseases and ill-health due to poor food safety.

A review on risk factors in street food practices in LMICs (Alimi, 2016) showed that there are three main categories of risk: environmental, chemical and micro-biological. This is in line with the themes that participants identified as concerns in our study, which included poor environmental sanitation, food contamination and adulteration, and food hygiene.

As was observed in other studies in LMICs (Alimi, 2016; Omari et al., 2018), our study found that among these three categories of risk factors, people are primarily concerned with food hygiene, i.e. microbial contamination of food and its health implications. Participants in our study did not provide in-depth information on the types of foods they would not eat because of food safety concerns, but rather just said that poor food hygiene would hinder healthy eating. A study conducted in urban Benin (Cotonou) amongst adolescents showed that food safety was a barrier to fruit and vegetable consumption both outside the home and within the school setting (Nago et al., 2012). Likewise, adolescents in urban Ethiopia also reported food safety as a major barrier to healthy dietary behaviours, showing a preference for packaged food (Trübswasser et al., 2020).

In our study, participants avoided certain food outlets and generally preferred eating home-made foods to avoid consuming adulterated and/or contaminated foods. The Food and Agriculture Organization of the United Nations (FAO) found that contamination and adulteration levels of food were ‘very high’ in street food outlets in Ghana and in conjunction with poor hygiene practices, increased the risk of diseases (FAO, 2016).

Our results on poor environmental sanitation in Ghana corroborate findings presented in the UN-HABITAT report for both Accra and Ho, highlighting an important problem of inadequate sanitation in the neighbourhoods (UN-HABITAT 2009a and 2009b). No comparable information was found for Kenya. The review by Alimi (2016) describes potential environmental pollutants (e.g. airborne chemicals in dusts; pollutants from moving vehicles; smoke; foul smell from accumulated waste; industrial effluents; rodents, insects and flies) that may contaminate foods and increase the transmission of diseases in the vending environment. Some of these were raised several times by our participants, e.g. insects and flies, offensive smell from accumulated waste and dust.

### 4.2 Strengths and limitations

The use of Photovoice provided an opportunity for a greater understanding of dietary behaviours and associated factors from participants’ perspective (emic rather than etic perspective). By placing emphasis on understanding phenomena from the perspective of participants, via placing cameras in their hands, their actual voices can be heard in the results of this study (Wang and Burris, 1997; Johnston, 2016). This is particularly important for groups that experience socio-economic disadvantage and/or marginalisation, such as included in this study. One of the limitations of the research included the choice of conducting individual in-depth interviews vs. the group discussions normally used in Photovoice (Wang and Burris, 1997). One of the main reasons for this was the fact that participants who were working were not able to easily come together in groups (particularly in the two big cities) and in some areas, safety issues made group meetings unethical. Whilst conducting group discussions may have given participants the confidence to discuss community issues and advocate for change together in a more powerful way, the individual approach allowed to capture a diversity of opinions as all participants had a voice (i.e. the loudest voice was not necessarily the most represented). Furthermore, participants could take part at times that were convenient for them meaning the most marginalised groups could be represented as they tend to have less time in this context.

A further strength of this study is that data have been collected in multiple cities from two countries therefore providing a range of geographical perspectives. However, the Photovoice activity represents the views of adolescent girls and boys and adult men and women living in selected deprived neighbourhoods in the three cities and therefore might not be representative of other communities.

In this study, the school and workplace settings were not represented as much as the home and neighbourhood levels. One reason why factors within these settings have not been discussed extensively could be that the study only had 13.4% of participants in education and 35.2% in work. Another reason could be that participants, in these settings, were either not allowed to take pictures or did not feel as comfortable to do so.

We held local photography exhibitions in the three neighbourhoods that were attended by a range of stakeholders including the study participants, local community members, NGOs, representatives from national government (Ministries of Health/Food and Agriculture), local government and the media. The exhibitions provided an opportunity for participants to see their work displayed in a public exhibition. This represented an important achievement for participants, who attended with a great sense of pride. These events also provided an opportunity to confirm whether our interpretation of the findings was accurate and then collaborate on designing recommendations sensitive to the local community. The photography exhibitions also linked participants to community leaders and officials who have the power to action change and in so doing, initiate a dialogue on how change can take place. One of the limitations of the exhibitions was that in line with what we had learned in our community engagement, the number of Photovoice participants who were able to attend was limited due to time constraints. Those that did attend were either of lower (i.e., those not in paid employment) or higher socio-economic status, meaning that we did not have representation from participants of middle socio-economic status. While this resulted in a lack of opinions from this group in the exhibitions, we did have representation from all socio-economic status groups in terms of their photographs. Food, drink and music provided additional incentives to come to the exhibition. However, taking time out – either during the week or the weekend – was not easy for study participants. We therefore recommend incentivising participants to come to the exhibition by considering the following: a free print of the photographs they selected for display, shuttle bus/taxi to the exhibition, child minder or entertainer for the children etc. The success of the exhibition relies on whether the participants of the study can attend the exhibition, so organisers need to develop strategies that would incentivise a visit and/or make it very easy to attend.

### 4.3 Policy implications

This study allowed the identification of policy options that would improve the physical food environment of participants living in the selected neighbourhoods in Accra, Ho and Nairobi, and ultimately, contribute to dietary health (i.e. diet diversity and food safety). Sustainable solutions that ensure financial and physical access to healthy food products (i.e. safe and nutritionally balanced) are required for socio-economically disadvantaged urban dwellers who currently suffer the greatest burden of poor nutrition and health.

With regards to economic access acting as a barrier to purchasing healthy foods, that are perceived as cost prohibitive, food pricing policies alongside social protection schemes, such as conditional cash transfers, would offer opportunities to reduce social inequalities (Eyles et al., 2012). These strategies may be an avenue to encourage healthy eating behaviours (Bortoletto-Martins et al., 2013; Fisher et al., 2017) and in turn reduce the risk of NR-NCDs. 10.13039/100014337Furthermore, given the positive implications that home gardening has on household food availability and accessibility of nutrient-rich food products (Talukder et al., 2000; Ali et al., 2008; Landon-Lane, 2011; Fraval et al., 2019), policies combined with programmes from local NGOs or women's groups could be developed to promote and support food insecure families to develop home gardens in urban poor settings where there is viable space to do this.

Food safety also requires urgent attention from local and national policy makers. Food policies and legislation need to be strengthened and their implementation enforced through market, food courts and other food places infrastructure improvements, food vendors training and regulation (Alimi and Workneh, 2016; Birgen et al., 2020). In Ghana, for example, food legislation is poorly regulated (Monney et al., 2014; Ababio and Lovatt, 2015) and there are no laws regulating street food vendors (FAO, 2016). Generally, food vendors have satisfactory knowledge of food safety, but this does not necessarily translate into good practice (Akabanda et al., 2017). In order to protect the community against unsafe practices and food-borne diseases, regulatory measures of the informal food sector, (e.g. code of practice and inspections), should be accompanied by measures to increase awareness of the risks of poor food safety (e.g. poor hand-washing, food handling, preparation and vending practices) amongst food vendors. In addition, infrastructural development and financial incentives are needed to support food vendors. To address food safety issues in the communities, a range of stakeholders need to be involved, from governments, food vendors, consumers’ associations, civil society groups to development partners (Alimi, 2016).

The emergence and expansion of supermarkets (i.e. ‘supermarketization’) in LMICs is supported by policymakers as often it is considered a remedy to food safety issues, through the implementation of private standards that would provide food safety guarantees (Reardon, 2006). In Asia, the replacement of wet markets by supermarkets has not proven successful, given that socio-economically disadvantaged populations do not have the means (financial or physical) to access supermarkets and these were often perceived as unfamiliar and unfriendly (Maruyama and Trung, 2007; Wertheim-Heck et al., 2019). Even when distance was not an issue, these were rarely visited by poor households in urban Vietnam (Wertheim-Heck et al., 2019). Overall, supermarkets contribute very little to poor urbanites' diets (Wertheim-Heck et al., 2019). The informal food sector in LMICs is essential for livelihoods, income generation and food security in the urban poor communities. Thus, replacing traditional markets by modern supermarkets may not be the solution as this may lead to unexpected consequences on livelihoods and nutrition. The discussions with participants at the photography exhibitions highlighted the need to retain informal food outlets as these are accessible to members of the community, but introduce and enforce legislation that vendors can realistically afford to implement and adhere to.

## 5 Conclusion

Ensuring food and nutrition security in urban poor settings remains a growing challenge in LMICs. The findings of this study increase our understanding of the wider contextual factors that need to be addressed in the urban, poor physical food environment, for adolescents and adults, to adopt healthier and safer dietary behaviours. Purchasing power, together with food safety and physical access to food, represent the key factors influencing participants’ dietary behaviours. Further studies are needed to assess the associations between perceived food safety and consumption of processed or EDNP foods (e.g. fried foods, packaged foods, sugar-sweetened beverages, sugary produtcs), as these may be perceived as safer by the consumer, and thus preferred, which could partly explain the increasing burden of NR-NCDs.

## Ethics statement

Ethical approval for the study was acquired by each institution involved in data collection: the Ghana Health Service Ethics Review Committee (GHS-ERC 07/09/16 and GHS-ERC 02/05/17) and the African Medical and Research Foundation (AMREF) (ESRC P365/2017) for Kenya. The ethical committee granted permission for photographs re-use in scientific outputs. Approvals from UK research institutions included: The University of Sheffield, Loughborough University (R17 -P142) and the University of Liverpool (1434 and 2288). Written informed consent was obtained from participants aged ≥18 years and assent from legal guardians of participants aged 13–17 yrs. A photograph release form was used to request consent to take photographs if a person's face was visible and participants consented to photographs being used in scientific outputs.

## Contributor statement

MH, PG, NB, MG, AL, FZ, EWK-M, EKR, MB, KM, RAk, RAr and RP designed the research study. All authors were involved in designing the data collection approach and tools. MNW, AT, SK, NC collected and transcribed the data. PG, RP, AT, SK, AI, FG and MNW analysed the data. RP wrote the first draft of the paper with critical input from AI, MH, PG and MNW. All authors reviewed the manuscript and approved the final version.

## Data availability

A data repository (DataSuds, part of the Dataverse Network) is used for this purpose. Metadata and tools used are found here for Ghana (https://doi.org/10.23708/XSACNA and https://doi.org/10.23708/V3EH6I) and here for Kenya (https://doi.org/10.23708/V3EH6I).

## Declaration of competing interest

None.

## Appendix A Supplementary data

Multimedia component1 Multimedia component1
