# Supplementary material for: Urban physical food environments drive dietary behaviours in Ghana and Kenya: A photovoice study
Source: Health Place. 2021 Sep;71:102647. doi: 10.1016/j.healthplace.2021.102647 (PMC8520917; doi:10.1016/j.healthplace.2021.102647)
Supplement: Multimedia component1 [file mmc1.docx]

**Supplementary Appendix 1: Selection of neighbourhoods in the three African cities**

In Accra, the selection of a neighbourhood was informed by the *Accra Poverty Mapping Exercise* (CHF International, 2010). Four areas were identified as being poverty endemic. Amongst these, Ga Mashie which is comprised of James Town and Ussher Town, was purposively selected as it was an area the research team could feasibly work in. A simple random sampling exercise was then applied, and James Town was selected as the neighbourhood of interest. In the city of Ho, the United Nations Human Settlements Programme (UN-HABITAT 2009) urban profiling report informed the selection of the study site. The report highlighted that 36% of the population lived in four poor areas within the city: Bankoe, Hliha, Ahoe and Dome (United Nations Human Settlements Programme, 2009). Amongst these four areas, Dome was then randomly selected. In Nairobi, we used data from the Kenya National Bureau of Statistics (Kenya National Bureau of Statistics, 2014) to identify the deprivation level of locations (wards), and randomly selected Makadara Constituency. Jericho, Bahati, Maringo, Hamza, Makongeni and Mbotela communities in Makadara were purposively selected as these were areas the research team could feasibly and safely work in.

**References**

CHF International. (2010). Accra Poverty Map: A guide to urban poverty reduction in Accra. Retrieved from: <https://www.globalcommunities.org/node/37186>

Kenya National Bureau of Statistics, Ministry of Health/Kenya, National AIDS Control Council/Kenya, Kenya Medical Research Institute, and National Council for Population and Development/Kenya. 2015. Kenya Demographic and Health Survey 2014. Rockville, MD, USA: Available at <http://dhsprogram.com/pubs/pdf/FR308/FR308.pdf>.

United Nations Human Settlements Programme. (2009). Ghana: Ho city profile. Retrieved from: <https://uni.unhabitat.org/wp-content/uploads/2014/07/Ghana-Ho-City-Profile.pdf>

**Supplementary Appendix 2: Quota sampling plan**

This study was based on two sister projects: DFC and TACLED. The DFC project was only conducted in Ghana (Accra and Ho) whilst the TACLED project was conducted in both Ghana (Accra only) and Kenya (Nairobi). The sampling strategy for these two projects is presented below.

**Table 2a: Quota sampling plan for the Photovoice activity in Accra (DFC project)**

|  | | | | | | | | | |
| --- | --- | --- | --- | --- | --- | --- | --- | --- | --- |
| **SES**  **Reproductive**  **Life Course** | Lowest SES | | | | Low to middle SES | | | | **N** |
| 13-14y (not pregnant or  lactating) | Not in work or education | | In work or education | | Not in work or education | | In work or education | | 8 |
|  | BMI<25 | BMI≥25 | BMI<25 | BMI≥25 | BMI<25 | BMI≥25 | BMI<25 | BMI≥25 |  |
| 15-49y (not pregnant or lactating) | Not in work or education | | In work or education | | Not in work or education | | In work or education | | 8 |
|  | BMI<25 | BMI≥25 | BMI<25 | BMI≥25 | BMI<25 | BMI≥25 | BMI<25 | BMI≥25 |  |
| 15-49y (pregnant) | Not in work or education | | In work or education | | Not in work or education | | In work or education | | 8 |
|  | BMI<25 | BMI≥25 | BMI<25 | BMI≥25 | BMI<25 | BMI≥25 | BMI<25 | BMI≥25 |  |
| 15-49y (lactating) | Not in work or education | | In work or education | | Not in work or education | | In work or education | | 8 |
|  | BMI<25 | BMI≥25 | BMI<25 | BMI≥25 | BMI<25 | BMI≥25 | BMI<25 | BMI≥25 |  |
| **Total sample** | | | | | | | | | **n=32** |

**Table 2b: Quota sampling plan for the Photovoice activity in Ho (DFC project)**

|  | | | | | | | | | |
| --- | --- | --- | --- | --- | --- | --- | --- | --- | --- |
| **SES**  **Reproductive**  **Life Course** | Lowest SES | | | | Low to middle SES | | | | **N** |
| 13-14y (not pregnant or  lactating) | Not in work or education | | In work or education | | Not in work or education | | In work or education | | 8 |
|  | BMI<25 | BMI≥25 | BMI<25 | BMI≥25 | BMI<25 | BMI≥25 | BMI<25 | BMI≥25 |  |
| 15-49y (not pregnant or lactating) | Not in work or education | | In work or education | | Not in work or education | | In work or education | | 8 |
|  | BMI<25 | BMI≥25 | BMI<25 | BMI≥25 | BMI<25 | BMI≥25 | BMI<25 | BMI≥25 |  |
| 15-49y (pregnant) | Not in work or education | | In work or education | | Not in work or education | | In work or education | | 8 |
|  | BMI<25 | BMI≥25 | BMI<25 | BMI≥25 | BMI<25 | BMI≥25 | BMI<25 | BMI≥25 |  |
| 15-49y (lactating) | Not in work or education | | In work or education | | Not in work or education | | In work or education | | 8 |
|  | BMI<25 | BMI≥25 | BMI<25 | BMI≥25 | BMI<25 | BMI≥25 | BMI<25 | BMI≥25 |  |
| **Total sample** | | | | | | | | | **n=32** |

**Table 2c: Quota sampling plan for the Photovoice activity in Accra (TACLED project)**

|  | | | | | | | | | |
| --- | --- | --- | --- | --- | --- | --- | --- | --- | --- |
| SES  age group | Lowest SES | | | | Low to middle SES | | | | **N** |
| 13-18y | Not in work or education | | In work or education | | Not in work or education | | In work or education | | 8 males |
|  | BMI<25 | BMI≥25 | BMI<25 | BMI≥25 | BMI<25 | BMI≥25 | BMI<25 | BMI≥25 |  |
| 19-49y | Not in work or education | | In work or education | | Not in work or education | | In work or education | | 8 males |
|  | BMI<25 | BMI≥25 | BMI<25 | BMI≥25 | BMI<25 | BMI≥25 | BMI<25 | BMI≥25 |  |
| ≥50y | Not in work or education | | In work or education | | Not in work or education | | In work or education | | 8 males  8 females |
|  | BMI<25 | BMI≥25 | BMI<25 | BMI≥25 | BMI<25 | BMI≥25 | BMI<25 | BMI≥25 |  |
| 2 participants per cell | | | | | | | | | |
| **Total sample** (n=8 females; 24 males) | | | | | | | | | **n=32** |

**Table 2d: Quota sampling plan for the Photovoice activity in Nairobi (TACLED project)**

|  | | | | | | | | | |
| --- | --- | --- | --- | --- | --- | --- | --- | --- | --- |
| SES  age group | Lowest SES | | | | Low to middle SES | | | | **N** |
| 13-18y | Not in work or education | | In work or education | | Not in work or education | | In work or education | | 8 males 8females |
|  | BMI<25 | BMI≥25 | BMI<25 | BMI≥25 | BMI<25 | BMI≥25 | BMI<25 | BMI≥25 |  |
| 19-49y | Not in work or education | | In work or education | | Not in work or education | | In work or education | | 8 males  8 females |
|  | BMI<25 | BMI≥25 | BMI<25 | BMI≥25 | BMI<25 | BMI≥25 | BMI<25 | BMI≥25 |  |
| ≥50y | Not in work or education | | In work or education | | Not in work or education | | In work or education | | 8 males  8 females |
|  | BMI<25 | BMI≥25 | BMI<25 | BMI≥25 | BMI<25 | BMI≥25 | BMI<25 | BMI≥25 |  |
| 2 participants per cell | | | | | | | | | |
| **Total sample** (n=24 females; 24 males) | | | | | | | | | **n=48** |

**Supplementary Appendix 3: Recruitment strategy**

To identify eligible participants, a screening questionnaire was administered to participants using electronic data capture (Samsung Galaxy tab-4), in order to obtain information relating to socio-demographic characteristics (i.e. place of residence; date of birth; weight and height for the calculation of body mass index; education; occupation and SES). In Ghana, SES was measured using the EquityTool which is a short, validated and country-specific tool to measure wealth (Chakraborty, Fry, Behl, & Longfield, 2016). Household SES scores were derived from 13 questions: ownership/facilities (colour television, refrigerator, video deck/DVD/VCD, bank account, electricity, wall clock, cabinet/cupboard, type of fuel used for cooking, household toilet facility, drinking water source, agricultural land, household floor and exterior walls material). Household scores were then compared to the average scores for urban Ghana and SES quintiles were subsequently derived. Participants were further classified into three groups: lowest SES (1st quintile); low to middle SES (2nd and 3rd quintiles) and high SES (4th and 5th quintiles). For this project, only participants in the 1^st^ and 2^nd^ tertiles, representing the lowest and low to middle SES respectively were selected. In Kenya, participants’ SES was derived from their total household expenditure. Based on the Kenya National bureau of Statistics’ classification of low/middle income households, those spending less than Ksh 23,670 per month were classified as lowest SES while those spending between Ksh 23,670 and Ksh 199,999 were classified as low to middle SES (Kenya National Bureau of Statistics, 2018).

The screening tools for both Ghana and Kenya were then imported into either CSPro version 6.3 or Survey CTO and were piloted using electronic data-capture. The screening tool was revised accordingly after piloting.

**References**

Chakraborty, N. M., Fry, K., Behl, R., & Longfield, K. (2016). Simplified asset indices to measure wealth and equity in health programs: A reliability and validity analysis using survey data from 16 countries. *Global Health Science and Practice*, *4*(1), 141–154. https://doi.org/10.9745/GHSP-D-15-00384

Kenya National Bureau of Statistics. (2018). Kenya Integrated Household Budget Survey 2015-2016. Retrieved from <http://statistics.knbs.or.ke/nada/index.php/catalog/88/study-description>

**Supplementary Appendix 4: Photovoice Interview Guide**

**Presenting the photovoice exercise to participants**

Good morning/afternoon (name of participant), we are here to discuss the photovoice activity we told you about during the 24hr recall data collection a few weeks ago.

What is Photovoice?

A process of collecting information and expressing issues and concerns through photos. Photovoice asks participants to use photographs to record aspects of their lives and experiences.

In this project ***(DIETARY TRANSITIONS IN AFRICAN CITIES)***, we will be asking you to take some pictures of the things that make you choose the foods and drinks you consume at home or elsewhere (e.g. people you relate with, your environment, and any other things). We are doing this, so that we can help the formulation of policies that improve diets.

We will give you a digital camera which you will use to take photographs on some topics we have identified in a topic list below.

The pictures you will take and the discussion we shall have with you on your pictures, will help us understand how our family members, friends, neighbours, the places we stay in and the places we go, affect or influence the foods we eat and drink.

Furthermore, these pictures will help us to find ways that could make women and adolescent girls in Ghana eat and drink more healthily. To this end we would like to share some of the photographs that you and the other women in this project take in an exhibition later in the project so that people who make decisions about food and drink in this neighbourhood can have a better idea of the things that influence the foods you eat and drink. Your pictures can therefore be powerful in making change!

This Photovoice exercise will be in two sessions.

Today, we will take you through the picture list and how to use the camera. We will show you how to turn the camera on/off, how to snap the pictures and how to check if the pictures are on the camera or not. Then we will go through the picture list.

There are some few things to note:

**Photo Ethics and Safety**

- If you take a picture and the face of person shows, you will need to ask permission from the person before the picture can be used. We will give you a ‘Photo Release’ Form to use to get permission.
- Though we want you to take these pictures for us, please ensure you are safe anytime you go out to take pictures.
- You can go with another person to take the pictures.
- Don’t do anything you wouldn’t usually do.
- Don’t go anywhere you wouldn’t usually go.
- Please do not enter into a person’s private space to take your pictures.
- Avoid disclosure of embarrassing facts about individuals through your pictures.
- Avoid taking pictures of people in such a way that leaves a negative and inaccurate impression about that person.

In the second part, we will come after 7 -10 days for the camera, have the pictures printed and then come back for a discussion on the pictures. During the 7-10 days, we will check in to see how you are doing and check the progress of the picture taking, as well as answer any questions you have with regards to the picture list or the camera we gave you.

Let's start with showing you how to use the camera and after, we will go through the picture list. Please let us know if you do not understand or something is not clear. We will take you through it till everything is clear.

**PICTURE LIST**

Please try and take at least one picture that shows each of the five headings/topics below

1)  A place where you eat food and/or drink

2) Something that makes eating healthy difficult for you (for example, time; price of food/drink; home; work; school; neighbourhood/community; restaurants and fast food outlets; supermarkets; convenience and corner store).

3) Something that makes eating healthy easy for you (for example, time; price of food/drink; home; work; school; neighbourhood/community; restaurants and fast food outlets; supermarkets; convenience and corner store).

4) something that influences what you eat in your neighbourhood/community (for example, income/money; home; work; school; neighbourhood/community; restaurants and fast food outlets; supermarkets; convenience and corner store).

5) A person that influences your food or drink choice in your area (i.e. family; friends; peers; other)

​​

**INTERVIEW PROTOCOL**

1. Ask Participants to return with SD card after 7-10 days
2. Print photographs for interview
3. Match photo numbers with photo topics for data storage and identification on the day of interview.

Remind participant

- 1. In this project, we are trying to understand the things that make you choose the foods and drinks you consume at home or elsewhere (e.g. people you relate with, your environment, and any other things).
  2. We are doing this, so that we can help the formulation of policies that improve diets.

1. Start qualitative interview (see guide below) using pictures as focus for the interview

**Photovoice: Qualitative Interview Questions**

- Can you pick the most important picture to you please?
  1. Why have you chosen this picture over other pictures you took to include?
  2. Can you tell me about what this picture shows? *(note: try and get a thorough description of the photos and make sure the participant has covered why the picture is important to understanding food choices in their daily life).*
  3. If you had to tell one sentence to tell the story of this picture to a person looking at it in an exhibition what would it be?
- Which picture do you want to talk about next? *(note: use this prompt for all other pictures left to discuss)*
- For each picture:
  1. Can you tell me about what this picture shows? *(try and get a thorough description of the photos).*
  2. Can you tell me why this picture is important to understanding your food choices in your daily life? *(ask this only if this has not been covered in the description of the picture above).*
- If you could choose a photo to appear in an exhibit to tell a story about the food and drink environment in your community (good or bad things), which one would it be and why? *(note: If photo contains a face, check a photo release form exists, if not, choose another picture)*
- Can you describe any photos you would like to have taken to tell us about food choices in your neighbourhood/community that you were not able to take? What stopped you being able to take these?
- Ask participant to say (or write) a short caption/sentence that best describes what their picture is showing *(note: only ask this if the participant did not come up with a caption/one sentence description when discussing this particular photo earlier).*

1. Debrief - What is your general feeling about this activity?
   1. Did you learn anything from this activity e.g. about what influences consumption of food and drink in your community?
   2. What was your experience with learning to handle the camera?
   3. Did you feel safe handling the camera outside?
   4. Do you have any suggestions for improving the process?

**Supplementary Appendix 5: Ethics and safety guidelines for Photovoice**

**Ethics and Safety**

- If you take a picture and the face of person shows, you will need to ask permission from the person before the picture can be used. We will give you a ‘Photo Release’ Form to use to get permission.
- Though we want you to take these pictures for us, please ensure you are safe anytime you go out to take pictures.
- You can go with another person to take the pictures.
- Don’t do anything you wouldn’t usually do.
- Don’t go anywhere you wouldn’t usually go.
- Please do not enter into a person’s private space to take your pictures.
- Avoid disclosure of embarrassing facts about individuals through your pictures.
- Avoid taking pictures of people in such a way that leaves a negative and inaccurate impression about that person.

**Supplementary Appendix 6: template codebook for Ghana for the physical-level environment**

| **Name of nodes** | **Description** |
| --- | --- |
| **Home** |  |
| Food environment |  |
| *Availability* |  |
| *Environmental sanitation* | e.g. the place surrounding the house e.g. bad smell from public toilet around homes sometimes deters them from eating. |
| *Facilities* | e.g. dining table, kitchen, TV e.g. It is rather unfortunate that we do not have dining hall here, but if it were to be my own house, I would have got a dining hall. e.g. eating in a comfortable space/in front of TV e.g. eating at home is relaxing, helps to not buy food from outside. |
| *Food hygiene* |  |
| *Food preparation methods* |  |
| *Food safety* | e.g. no adulteration of foods; natural foods without additives |
| *Quality and Freshness of food* |  |
| Housing conditions |  |
| **Neighbourhood-community food environment** |  |
| Aroma of food |  |
| Availability (type of outlet, food attributes, ease of dispensing the food items) | e.g. the shop/restaurant is near the house and easily accessible, it makes it easy to get food there and break the monotony of cooking in the house e.g. favourite foods easily available in outlets within walking distance e.g milk ATMs allow one to buy what they can afford-no size or amount restrictions. e.g. larger market has more variety that smaller stands e.g. unlikable foods sold frequently in the area (offals) |
| Convenience, opening times | e.g. convenient opening times/days (open early and closed late) e.g. convenient place to sit at the outlet if you prefer to eat there e.g. food outlet within a structure rather than open air e.g. those with quickly available foods |
| Environmental sanitation | e.g. the place surrounding the outlet e.g. not located close to open sewers etc. |
| Facilities and services |  |
| Financial access (i.e. cost) | e.g. cheap/easily affordable and pocket friendly e.g. larger market cheaper than smaller stands-tomatoes are 4 for 10 vs. 3 for 25, items that are available to all classes of people e.g. poverty will not allow you to eat a balanced or well cooked meal e.g. food is available in small and affordable quantities. You use little money but get satisfied, you don’t have to spend a lot of your money. |
| Food adulteration & contamination |  |
| Food display, appearance of food and advertising |  |
| Food hygiene | e.g. food itself and people handling food e.g. hygiene standards e.g. clean, well packaged, well stored (so that dust cannot get in), wearing protective gear like a safety coat, gloves and a hair cover e.g. the place where the food is cooked is clean and they observe hygiene, wearing protective clothing while cooking |
| Food preparation methods |  |
| Food taste |  |
| Food waste |  |
| Physical access (i.e. distance to food outlet) |  |
| Quality and freshness of food |  |
| Regulations |  |
| Safety and security around food outlets |  |
| **School food environment** |  |
| Availability (i.e. type of outlets; school canteen) |  |
| Convenience |  |
| Environmental sanitation | e.g. the place surrounding the outlet |
| Financial access (i.e. cost) |  |
| Food adulteration & contamination |  |
| Food display and advertising |  |
| Food hygiene | e.g. food itself and people handling food |
| Food preparation method |  |
| Food taste |  |
| Food waste |  |
| Physical access (i.e. distance to food outlet) |  |
| Quality and Freshness of Food |  |
| Regulations |  |
| Safety and security around the food outlet |  |
| **Work food environment** |  |
| Availability (i.e. type of outlets; food available) | e.g. when selling food is their business they get to take home some of the produce |
| Convenience | e.g. convenient opening times, convenient place to sit at the outlet if you prefer to eat there |
| Environmental sanitation | e.g. the place surrounding the outlet |
| Facilities and services |  |
| Financial access (i.e. cost) |  |
| Food adulteration & contamination |  |
| Food display and advertising |  |
| Food hygiene | e.g. food itself and people handling food e.g. hygiene standards |
| Food preparation methods |  |
| Food taste |  |
| Food waste |  |
| Physical access (i.e. distance to food outlet) | e.g. choices available close to their place of work such that they do not take too long to return |
| Quality and Freshness of Food |  |
| Regulations |  |
| Safety and security around the food outlet |  |
| **Pictures** |  |
| A person that influences your food or drink choice in your area |  |
| A place where you eat food and drink |  |
| Something that influences what you eat in your area |  |
| Something that makes eating healthy difficult for you |  |
| Something that makes eating healthy easy for you |  |

Note: This codebook only includes the nodes and sub-nodes for the physical-level of the socio-ecological framework we used to guide the analysis. However, the full codebook covers all four levels of the socio-ecological framework: i. individual (e.g. preferences, knowledge, socio-demographic characteristics); social (e.g. family, friends and peers); physical (refers to environments in which people eat or source food, including the home, workplace, schools, restaurants and supermarkets) and macro (e.g. food marketing, food production and distribution systems).

**Supplementary Appendix 7: Examples of quotes and photographs from the work food environment**

| ***Sub-theme 1: Availability*** | | |
| --- | --- | --- |
| Accra | *‘…that is the work that I do. I smoke fish and distribute to women to sell at the market. This makes eating easy for me and I don’t go hungry. Sometimes If I am hungry, no matter where I am, whether at the seaside or market, I just take some of the fish and eat. I will drink water afterwards and that will take me for the day without eating anything else.’ [Female, 47 years, lowest SES, A39]* | 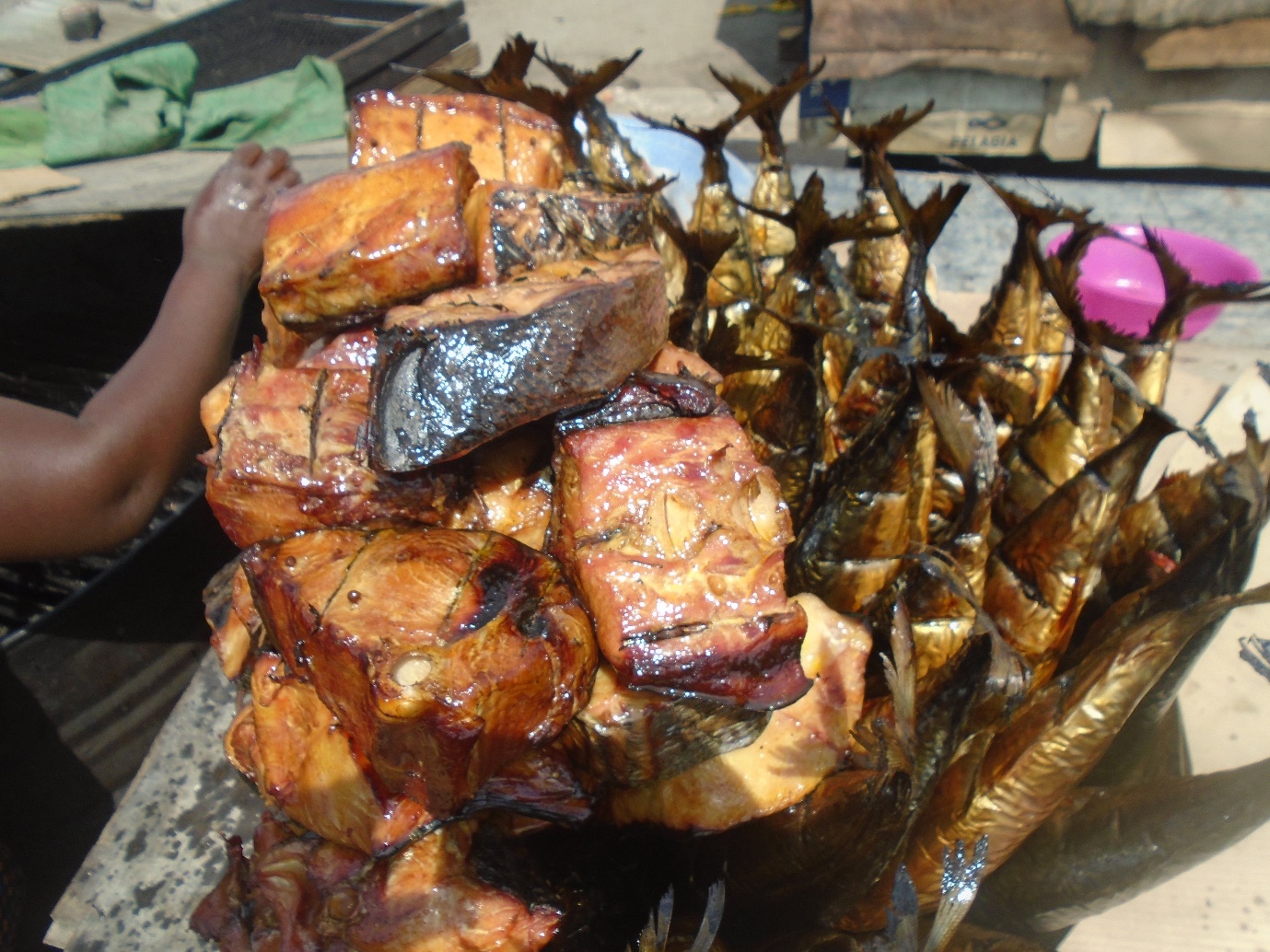 |
| Ho | *‘In the morning for breakfast as they give us the money to go and buy porridge, and the boss will see that we have bought the porridge, and then for lunch some rice… but we don’t go according to it. The boss can just come, I don’t have money for any jollof for staff so cook banku. And when he is not there, the kitchen staff may say yesterday we cooked banku so you people will still eat the banku… you don’t make your choice of soup when you come too.’*  *[Female, 42 years, low to middle SES, H32]* | 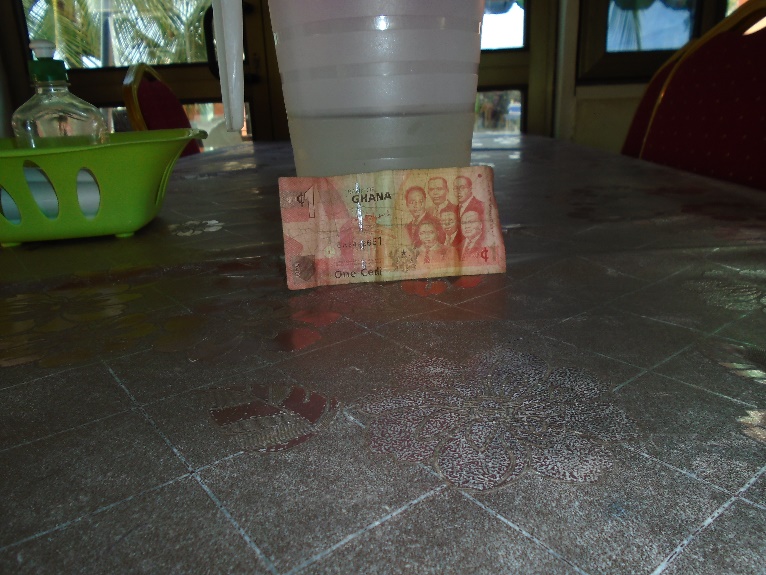 |
| ***Sub-theme 2: Convenience*** | | |
| Accra | *‘We normally buy the food far from the working site. The place where we buy the food is very far that you cannot go back and go and buy the food and come back. You will have spent about 30 minutes so we buy the food and we bring it to the site early in the morning when we are going to the site. After we are done working and it is afternoon then we eat.’ [Male, 26 years, low to middle SES, A19]* | 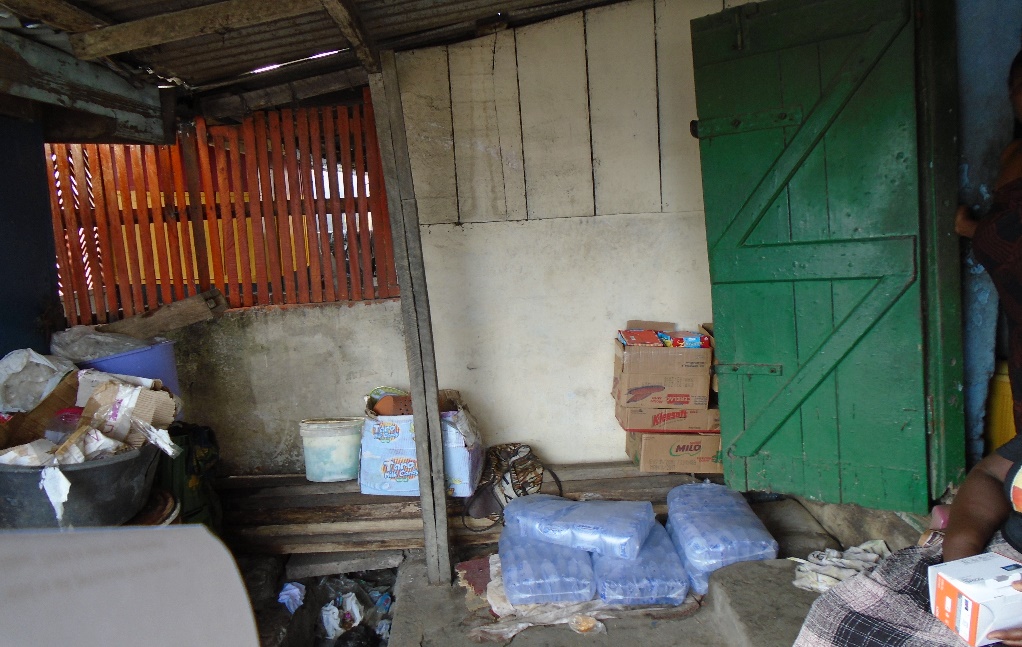 |
| Ho | *‘If there is no time, you won’t get the chance to eat. So if I have work to do and I am hungry, and I also have to teach the kids, then it means I will not have the time to eat. So, it shows that, it is the work that I do that does not make me have the time to eat.’* *[Female, 30 years, low to middle SES, H10]* | 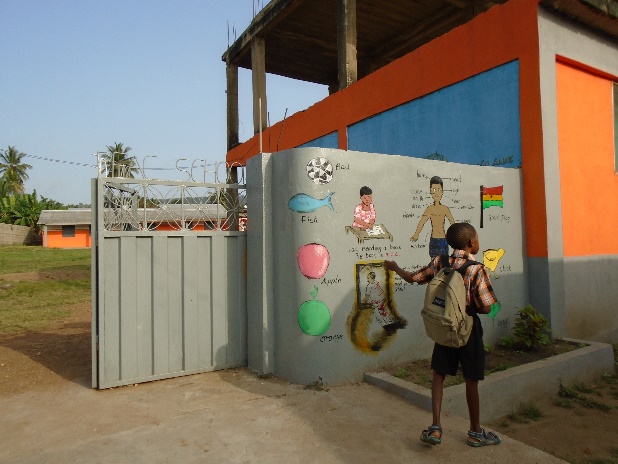 |
| Nairobi | *‘It is a place (international airport) that is very convenient for me on the side of getting food and getting, That photo is at a parking where we park our vehicles as we wait for visitors that we deal with as tours and travel.’ [Male, 55 years, lowest SES, N35]* | 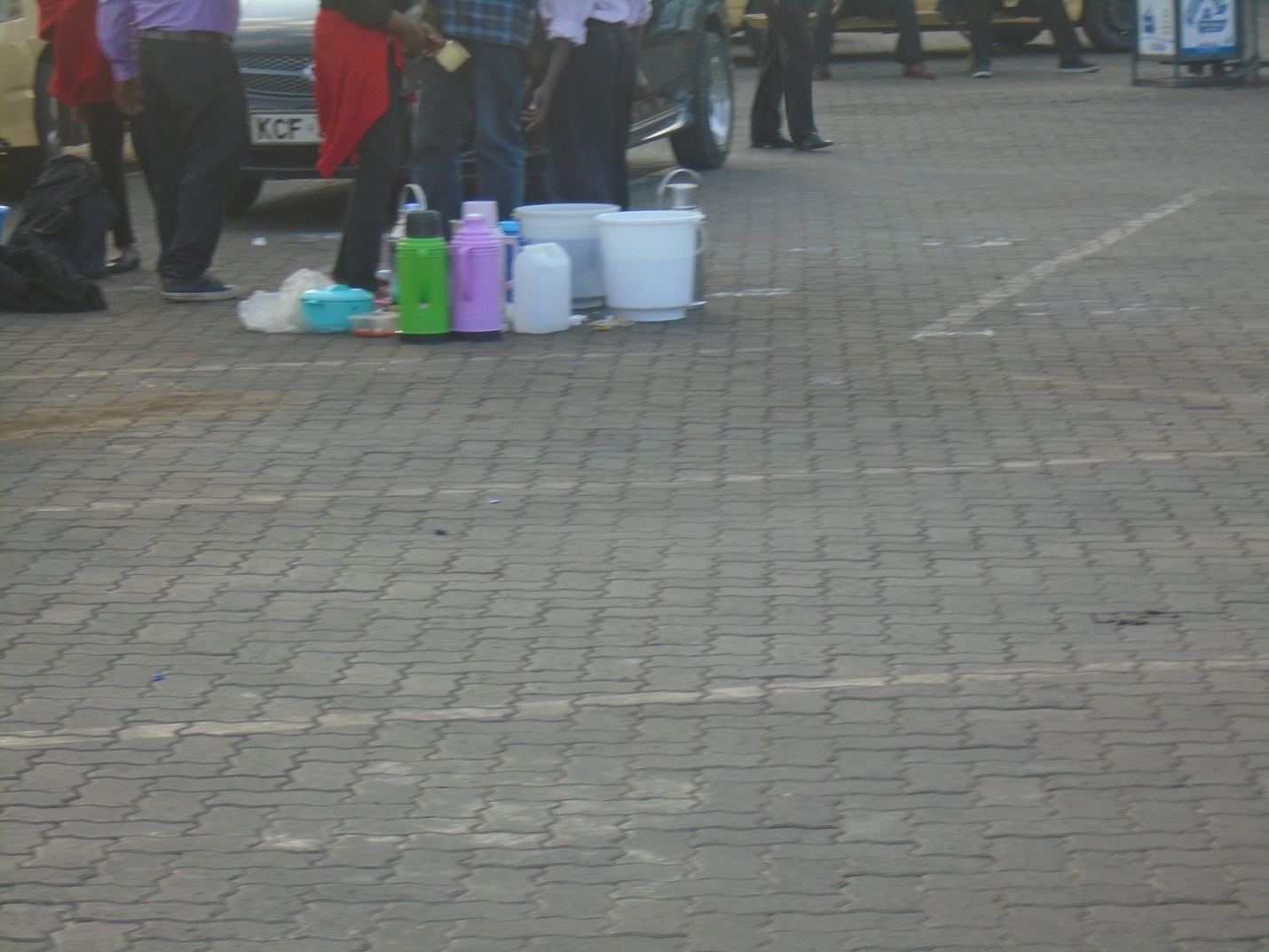 |
| ***Sub-theme 3: Facilities and services*** | | |
| Accra | *‘R: The place is a new building that we just sit on the floor or the bench that we do work on to eat. I: Okay but does this condition affect the way you eat and you your food environment? R: Yes, because you don’t feel comfortable when eating without a chair and table.’ [Male, 26 years, low to middle SES, A19]* | 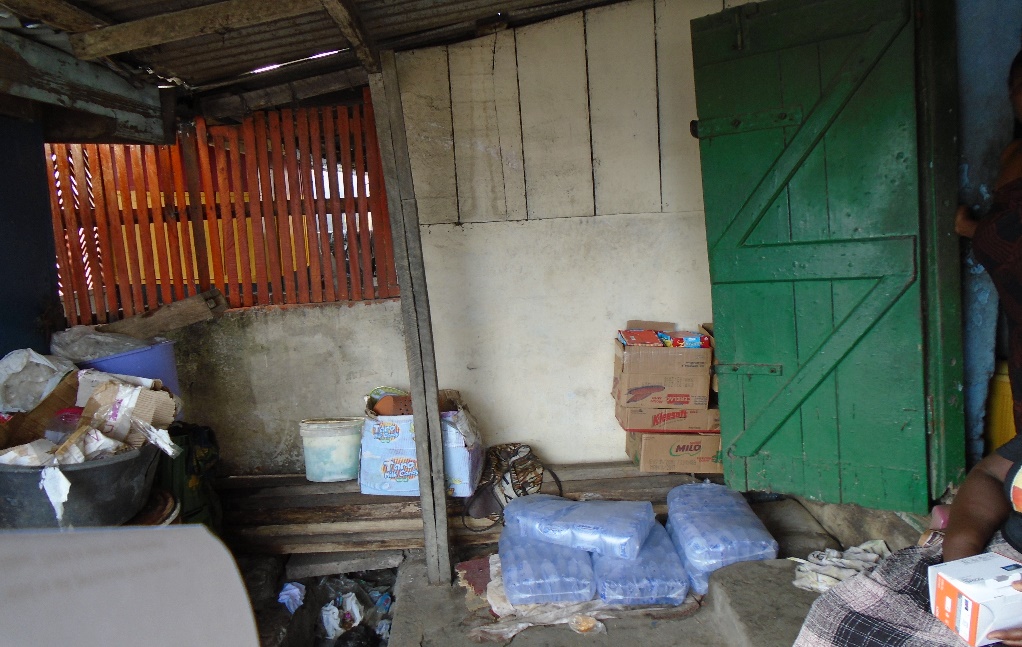 |
| Ho | *‘We don’t have a particular place for eating, so we sit here or we sit outside. You sit inside and you will see the customers coming from the outside. We have it, we microwaves, about three, but if you use it, you will buy prepaid. Or they will deduct it from your salary.’* [*Female, 42 years, low to middle SES, H32*] | 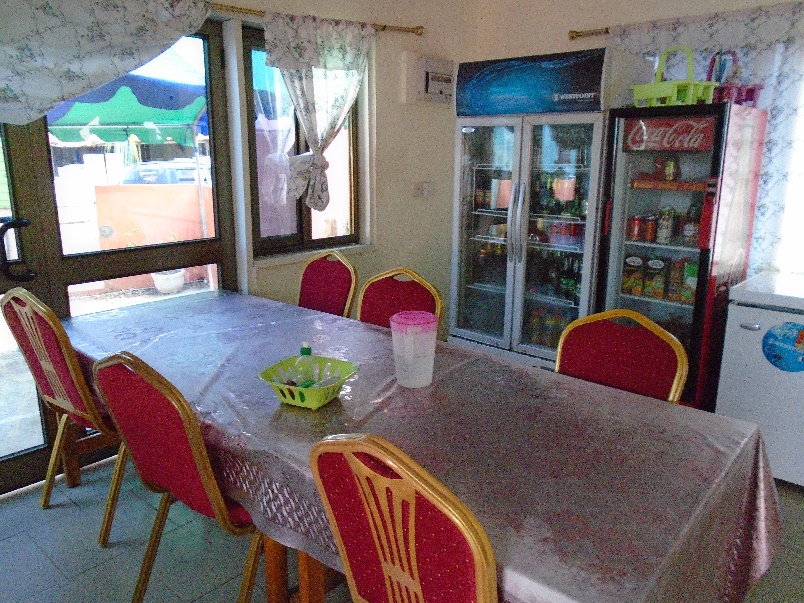 |
| ***Sub-theme 4: Food hygiene and environmental sanitation*** | | |
| Ho | *‘I was eating before the girl came to buy, so I had to leave the food there. And you can see I don’t have anything like a cover or something. That’s why I am eating the food in a rubber. Sometimes I use the rubber to cover it, sometimes too flies can penetrate through the rubber and enter the food. So before I will finish attending to the customer, I will lose appetite for the food and then I will have to throw it away.’ [Female, 18 years, low to middle SES, H18]* | 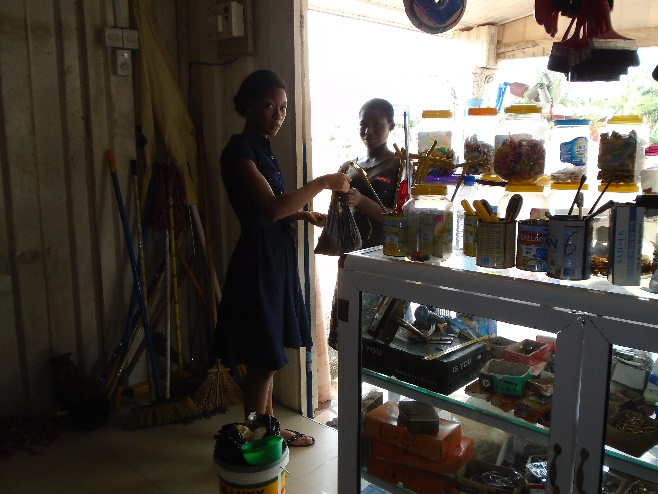 |
| ***Sub-theme 5: Economic access*** | | |
| Ho | *‘…the things they sell are expensive. They do cook healthy stuff, but the money to buy the things is not there and since they are expensive you can’t buy it.’* *[Female, 38 years, low to middle SES, H27]* | 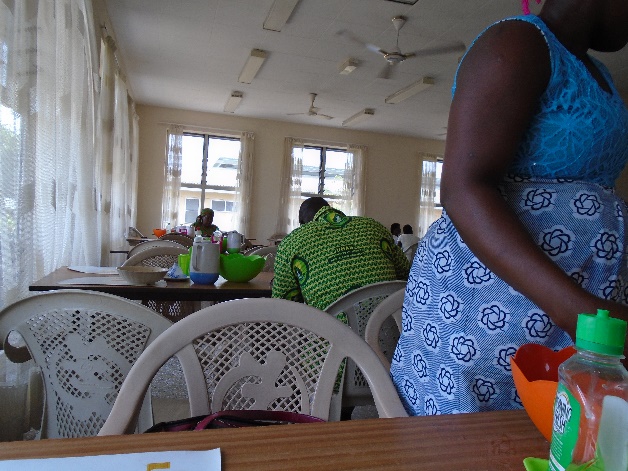 |

**Supplementary Appendix 8: Examples of quotes and photographs from the school food environment**

| ***Sub-theme 1: Availability*** | | |
| --- | --- | --- |
| Accra | *‘We are fed at the school canteen and it is compulsory that we pay and we are served with the food. So we are not allowed to buy food outside. We pay 5 cedis everyday for the canteen food. If in case you dislike the food provided at the canteen, you can go and report to the headmaster and he will talk to the caterer’ [Female, 14 years, low SES, A41]* | |
| Ho | *‘This picture is a poly tank filled with water and we students fetch water from it to drink when we are thirsty.’ [Female, 13 years, low to middle SES, H29]* | 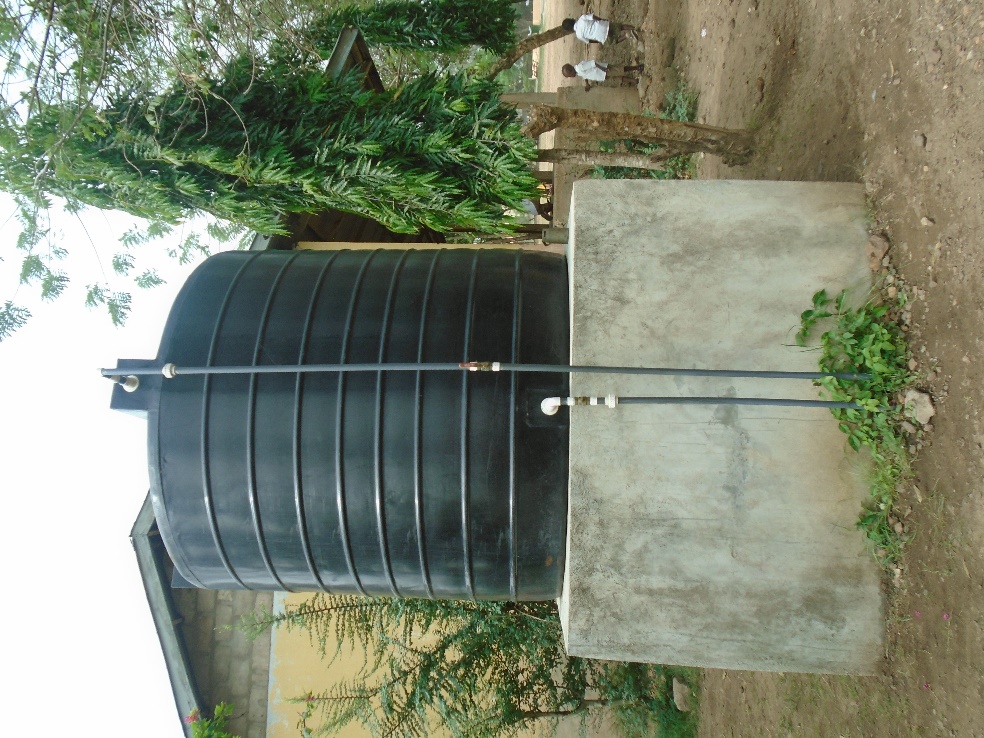 |
| ***Sub-theme 2: Convenience*** | | |
| Accra | *‘I would say the time for break is also not enough. We get two breaks when we go to school and each break is for about 15minutes. The first break is at 9:45am and then 12:50 in the afternoon. School starts at 7 and we close at 3 but we have compulsory afternoon classes so we close at 4pm. Sometimes it is only when I get home that I am able to eat well.’ [Female, 13 years, low to middle SES, A45]* | |
| ***Sub-theme 3: Food hygiene and environmental sanitation*** | | |
| Accra | ‘Sometimes the bowls in the school are not clean so it makes me buy food in a rubber bag to eat.’ *[Female, 13 years, low to middle SES, A45]* | |
| Ho | *‘When you get to the school, this is at the roadside and we buy from there. There is one on the school compound but I don’t buy from there because they have not kept the place well. And the place I thought was good and I have been buying food from, this is how to looks. It is even worse than the one on the school compound.’ [Female, 18 years, low SES, H4]* | *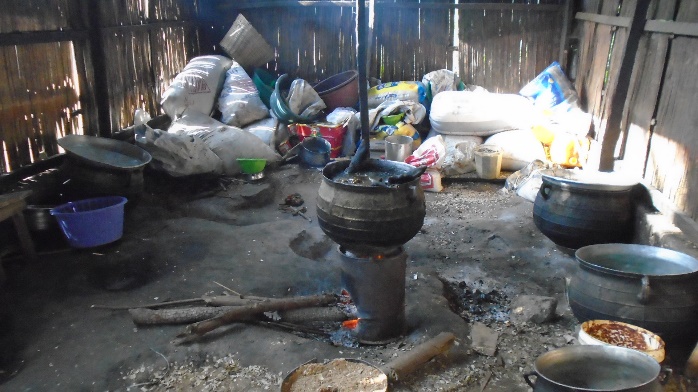* |
| ***Sub-theme 4: Economic access*** | | |
| Accra | *‘I am able to eat easily at school. The food is prepared nicely but there is one woman whose food is not well prepared but people still buy because it is not expensive. Someone also sells biscuits and sweets which I like. They are not expensive.’ [Female, 13 years, low to middle SES, A56]* | |
